# Effect of sorafenib starting dose and dose intensity on survival in patients with hepatocellular carcinoma: Results from a Canadian Multicenter Database

**DOI:** 10.1002/cam4.3228

**Published:** 2020-06-11

**Authors:** Mohammed A. Alghamdi, Carla P. Amaro, Richard Lee‐Ying, Hao‐Wen Sim, Haider Samwi, Kelvin K. Chan, Jennifer J. Knox, Yoo‐Joung Ko, Mina Swiha, Eugene Batuyong, Adriana Romagnino, Winson Y. Cheung, Vincent C. Tam, the Hepatocellular Carcinoma ‐ Cancer Health Outcomes Research Database (HCC ‐ CHORD) Consortium

**Affiliations:** ^1^ Tom Baker Cancer Centre University of Calgary Calgary AB Canada; ^2^ College of Medicine King Saud University Riyadh Saudi Arabia; ^3^ Princess Margaret Cancer Centre University of Toronto Toronto ON Canada; ^4^ St. Michael's Hospital University of Toronto Toronto ON Canada; ^5^ Sunnybrook Odette Cancer Centre Toronto ON Canada; ^6^ Canadian Centre for Applied Research in Cancer Control Toronto ON Canada; ^7^ University of Western Ontario London ON Canada; ^8^ Menoufia University Shebin El Kom Egypt

**Keywords:** discontinuation rate, dose intensity, hepatocellular carcinoma, sorafenib, starting dose

## Abstract

**Background:**

Sorafenib has been shown to improve survival in patients with advanced hepatocellular carcinoma (HCC), however, full dose can be difficult to tolerate. The aim of this study was to determine whether sorafenib starting dose and mean dose intensity affect survival.

**Methods:**

Patients treated with sorafenib for HCC from January 2008 to July 2016 in several Canadian provinces were included and retrospectively analyzed. The primary end point was overall survival (OS) of patients starting on sorafenib full dose compared to reduced dose. Secondary analysis compared OS with different mean dose‐intensity groups. Survival outcomes were assessed with Kaplan‐Meier curves and Cox proportional hazards models. A propensity score analysis was performed to account for treatment bias and confounding.

**Results:**

Of 681 patients included, sorafenib was started at full dose in 289 patients (42%). Median survival for starting full and reduced dose was 9.4 months and 8.9 months (*P* = .15) respectively. After propensity score matching and adjusting for potential confounders there was still no difference in survival (HR 0.8, 95% CI, 0.61‐1.06, *P* = .12). Almost half of the patients (45%) received a dose intensity < 50%. Median survival for mean dose intensity > 75%, 50%‐75%, and < 50% were 9.5 months, 12.9 months, and 7.1 months (*P* = .005) respectively. In multivariable models, starting dose(HR 1.16, 95% CI 0.93‐1.44, *P* = .180) and mean dose intensity were not associated with survival.

**Conclusions:**

Starting HCC patients on a reduced dose of sorafenib compared to full dose may not compromise survival. Mean dose‐intensity of sorafenib may also not affect survival.

## Graphical abstract

Starting dose and dose intensity of sorafenib does not impact mortality in patients with hepatocellular carcinoma. They also do not appear to affect discontinuation rates due to toxicity.

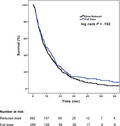

## 1 INTRODUCTION

Hepatocellular carcinoma (HCC) is the sixth most commonly diagnosed cancer and the fourth most common cause of cancer‐related death in the world.^1^ In Canada and the United States, the incidence of HCC and related‐deaths remains low, but there has been a significant increase over the past two decades.^2^, ^3^

Despite recent advances in systemic treatment for HCC, sorafenib remains a first‐line treatment option for advanced HCC. Two large randomized clinical trials, the SHARP and Asia‐Pacific trials, demonstrated that sorafenib improves overall survival (OS) compared to placebo in patients with advanced HCC.^4^, ^5^ Sorafenib remained a standard of care in the first‐line treatment of advanced HCC even after a recent study showed that lenvatinib is noninferior to sorafenib for survival in these patients.^6^ Many patients cannot tolerate the full dose of sorafenib due to adverse events, including: fatigue, diarrhea and hand‐foot skin reaction.^7^, ^8^, ^9^ In clinical practice some physicians start sorafenib at a lower dose to assess tolerance and hopefully keep patients on treatment.^10^ If the lower dose is well‐tolerated, then the dose is escalated.

Two smaller studies of Japanese HCC patients compared starting sorafenib at full (800 mg/d) vs half (400 mg/d) dose and did not find a significant difference in progression‐free survival (PFS) or OS.^11^, ^12^ A large retrospective study of almost exclusively male patients (99%) in the United Statestreatedat Veterans Affairs hospitals examined starting sorafenib at full dose (800mg/day) compared to reduced dosage (<800 mg/d).^13^Their results also supported no OS difference between starting HCC patients at full dose or reduced dose sorafenib.

Since dosing of drugs is determined by maximal tolerated dose in a majority of patients in phase I trials, the suggested starting dose of these drugs may not reflect the minimum effective dose required to treat the cancer. When patients have toxicities from treatment, oncologists are often asked whether reducing the dose will alter the efficacy of the treatment. The answer to this question is usually unclear.

The purpose of this study was to examine whether sorafenib starting dose affects survival in a large and more heterogeneous population of HCC patients. Furthermore, we examined mean dose intensity of sorafenib over the full course of first‐line systemic treatment since we hypothesized that this may have a greater effect on survival than starting dose.

## 2 METHODS

Patients treated with at least one dose of sorafenib between January 2008 to July 2016 at any of the 12 cancer centers in the Canadian provinces of British Columbia and Alberta, as well as 2 large centers in Toronto, Ontario (Princess Margaret Cancer Centre and Sunnybrook Odette Cancer Centre) were included in this study. The coordinating centre was Tom Baker Cancer Centre, University of Calgary (Research Ethics Board approval: HREBA.CC‐15‐0042). Research ethics approval was also obtained from the collaborating institutions. Canada has a government‐funded health care system that is provincially administered. Academic and community cancer centers are represented in the data by British Columbia and Alberta, while Princess Margaret Cancer Centre and Sunnybrook Odette Cancer Centre are both academic institutions.

All patients included for analysis had a diagnosis of HCC confirmed pathologically or with a combination of radiographic and biochemical features. These noninvasive HCC diagnostic criteria include: presence of liver cirrhosis, >2 cm focal HCC lesion with arterial phase hypervascularization, and alpha fetoprotein (AFP) level > 400 ng/mL. Patients who did not receive at least one dose of sorafenib were excluded.

### 2.1 Data collection

Patient demographics including age, gender, ethnicity (based on the last name and/or documented ethnicity),^14^ Eastern Cooperative Oncology Group (ECOG) performance status, underlying liver disease etiology and Child‐Pugh score were collected. Tumor‐related characteristics (eg stage, AFP) and pre‐sorafenib treatment characteristics were also collected. Sorafenib starting dose and mean dose intensity were collected and analyzed using pharmacy prescriptions orders and filled prescriptions and corroborated by clinical notes. Toxicities and reason for discontinuation of sorafenib were also collected. Specific toxicities were assessed from the clinical records, including hand‐foot skin reaction, elevated blood pressure, nausea, diarrhea, fatigue, mucositis, headache, alopecia, weight loss, and decreased appetite. Grades of these toxicities could not be accurately assessed due to lack of details in most treating physician notes.

The data were manually extracted from electronic patient records and pharmacy databases at each of the sites by the following individuals: British Columbia (RLY, HS), Alberta (MS, MA), Princess Margaret Cancer Centre (HWS), and Sunnybrook Odette Cancer Centre (AR, YJK). The data were collected on identical Excel spreadsheets (version 15.0; Microsoft Corporation) and subsequently merged for analysis. Missing data and inconsistencies were identified by EB and resolved by the data collector(s) at each site.

### 2.2 Study groups

Starting dose of sorafenib was classified as full dose if the patient received 800 mg per day and reduced dose if the starting dose was less than 800 mg per day. Mean dose intensity was calculated as the sum of all daily doses of sorafenib in milligrams taken by a patient divided by the full daily dose(800 mg per day) multiplied by the total number of days on sorafenib. The mean dose intensity was divided into three categories: >75% (>600 mg/d), 50%‐75% (400 mg‐600 mg/d), and <50% (<400 mg/d).

### 2.3 Outcomes

With respect to both the starting dose of sorafenib and mean dose‐intensity analyses, OS was the primary outcome. OS was calculated in months from the start date of sorafenib to date of death with censoring at last follow‐up. Exploratory outcomes of interest included: response rate (RR), disease control rate (response + stable disease) (DCR), percentage of patients stopping sorafenib due to adverse events, duration of treatment and proportional of patients who had dose modifications after the start of treatment. Comparison of adverse events between groups was performed as a univariate analysis. Response to treatment was assessed according to Response Evaluation Criteria in Solid Tumors (RECIST) 1.1 criteria based on imaging reports from local radiologists and if unclear, direct examination of the computed tomography (CT)images.

### 2.4 Statistical analysis

Descriptive statistics were calculated and categorical variables were compared using the chi‐square test. Continuous variables were compared using the Mann‐Whitney U and Kruskal‐Walis tests, specifically to compare the duration of sorafenib use. Kaplan‐Meier (KM) curves for OS were generated and compared using the log‐rank test. A Cox‐proportional hazard model was constructed with starting dose, dose intensity, and relevant clinical and pathologic factors to assess their impact on survival. Due to the risk of multicollinearity with starting dose and dose intensity, these variables were also analyzed independently in the model. As results were similar, there was limited evidence of multicollinearity, so the full model with both factors is presented. In exploratory analyses, such as rates of adverse events between groups, proportions were compared using the chi‐square test.

To further account for treatment bias and confounding, a matched propensity score analysis was performed for the comparison of starting doses of sorafenib and for the comparison of mean dose‐intensity. The propensity score was estimated using a logistic regression model based on starting dose. Variables used to construct the propensity score were age, gender, ethnicity, liver disease etiology, ECOG performance status, Child‐Pugh, TNM stage, portal vein thrombosis (PVT), tumor extension, number of tumors, prior treatments number and type (liver resection, ablation, transarterial chemoembolization (TACE), transarterial radioembolization (TARE), stereotactic body radiation therapy (SBRT), alcohol injection, transplant).

A propensity score matching analysis was performed using a macro program (%GMATCH) performing greedy matching.^15^ In this propensity score matching technique, calipers of width equal to 0.2 of the standard deviation of the logit of the propensity score was used in the %GMATCH macro. A standard difference of less than 0.1 was used as indicative of negligible difference of baseline covariates between the original and matched sample.

An inverse probability of treatment weight (IPTW) analysis using the propensity score as weights was also performed to account for the potential imbalance between groups to assess for robustness of findings. A standard difference of less than 0.1 was also used to assess balance between groups after weighting. OS as the primary outcome was assessed in the matched and weighted samples.

The propensity score analyses were performed using SAS 9.3. All other analyses were performed using IBM SPSS Statistics version 23.0 (IBM Corp). Results were considered statistically significant when *P*‐value < .05. Analyses were not adjusted for multiplicity.

## 3 RESULTS

### 3.1 Baseline characteristics

A total of 681 HCC patients treated with sorafenib were included in our multicenter database. The median age in our cohort was 64 years, 80% of patients were males and 37% were East‐Asian. The most common risk factors for HCC were hepatitis B (33%) and hepatitis C (29%) followed by alcohol (13%). As expected, most patients (86%) had Childs Pugh A liver function, although 13% were Childs Pugh B. Most patients had at least one prior local treatment for their HCC (69%), with the most common being TACE (33%), followed by liver resection (24%) and radiofrequency ablation (22%). Initial performance status was ECOG 0 in 30% and ECOG 1 in 58% of patients. AFP was elevated in 78% of patients within one month before starting sorafenib. Mean starting AFP was 19 836 ug/L. BCLC stage was B in 6% and C in 91%. Metastatic disease was present in 47% of the patients at baseline.

The median follow‐up period after sorafenib therapy was 6 months. The median overall survival was 9.1 months for the whole cohort.

### 3.2 Starting dose comparison

Table 1 shows the patient characteristics of those starting at full dose compared to reduced dose. Overall, only 42% started with full dose sorafenib while 58% started with a reduced dose. Of the 223 patients who were ≥ 70 years of age, 69% were started on a reduced dose compared to 52% in younger patients < 70 years of age (*P* = .19). Patients who were of East‐Asian ethnicity and those with any prior localized treatment, especially radiofrequency ablation or TACE, were more likely to have started on a reduced dose of sorafenib.

**TABLE 1:** Characteristics of patients comparing starting full dose to reduced dose

Patient characteristic	Dose reduced n (%) N = 392	Full dose n (%) N = 289	*P*
Age, years, mean	65	62	.186
<70	239 (61%)	219 (76%)	
≥70	153 (39%)	70 (24%)	
Gender			.054
Male	305 (78%)	242 (84%)	
Female	87 (22%)	47 (16%)	
Ethnicity			
East‐Asian	158 (40%)	92 (32%)	.023
Other	234 (60%)	197 (68%)	
Liver disease etiology			.74
None	66 (25%)	51 (18%)	
Hepatitis B	133 (34%)	89 (31%)	
Hepatitis C	117 (30%)	78 (27%)	
Alcohol	47 (12%)	45 (15%)	
NASH	18 (4%)	14 (5%)	
Other	11 (3%)	12 (4%)	
Any prior treatment	287 (73%)	183 (63%)	.006
Liver resection	89 (23%)	74 (26%)	.38
Radiofrequency Ablation	98 (25%)	50 (17%)	.016
Alcohol Injection	15 (4%)	9 (3%)	.618
Bland Embolization	14 (3.6%)	7 (2%)	.391
TACE	156 (40%)	71 (25%)	<.001
TARE	8 (2%)	14 (5%)	.041
Baseline AFP level			.672
>400	165 (42%)	123 (43%)	
≤400	204 (52%)	142 (49%)	
Unknown	23 (6%)	24 (8%)	
Baseline ECOG performance status			.258
0‐1	344 (88%)	251 (87%)	
2	40 (10%)	34 (12%)	
3‐4	5 (1%)	3 (1%)	
Baseline liver function (Child‐Pugh)			.263
A	330 (84%)	255 (88%)	
B	60 (15%)	32 (11%)	
C	0	1 (<1%)	
Baseline metastatic disease			.795
Yes	185 (48%)	135 (47%)	

Abbreviations: AFP, alpha fetoprotein; ECOG, Eastern Cooperative Oncology Group; NASH, non‐alcoholic steatohepatitis; TACE, trans‐arterial chemoembolization; TARE, trans‐arterial radioembolization.

**FIGURE 1:**
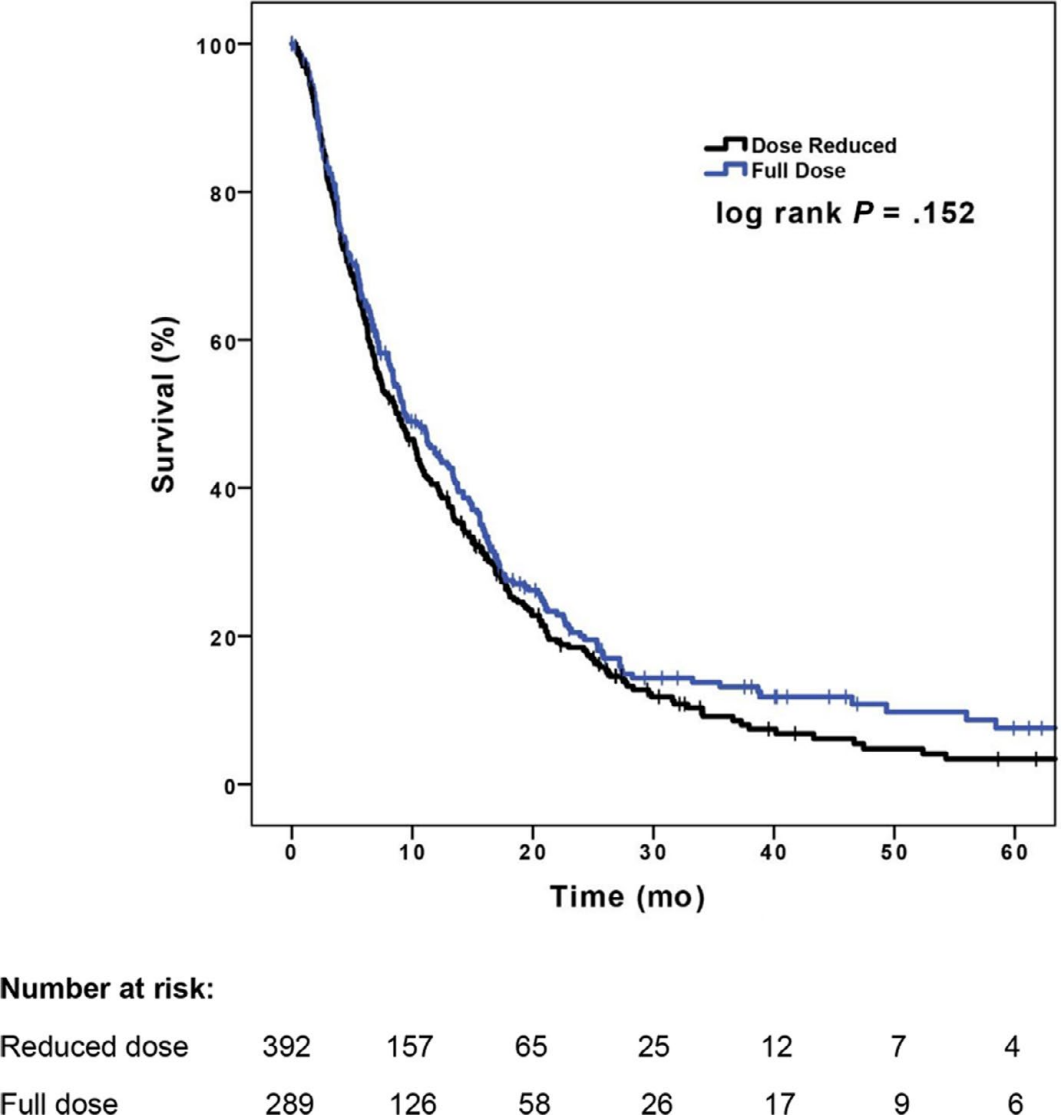
Kaplan‐Meier survival curves for starting full dose vs reduced dose

For those who started full dose of sorafenib and reduced dose, the median OS was 9.4 months (95% CI, 7.4‐11.4 months) and 8.9 months (95% CI, 7.3‐10.4 months) respectively. The Kaplan‐Meier curves are shown in Figure 1. There was no statistically significant difference in survival between these two groups (*P* = .15), and any estimated differences were too small to be clinically meaningful.

### 3.3 Mean dose intensity comparison

Table 2 shows the patient characteristics of those treated at different mean dose intensities of sorafenib. Over the course of their treatment, 31% of patients received sorafenib at a mean dose intensity of >75%, 24% at a mean dose intensity 50%‐75% and 45% at a mean dose intensity < 50%. Only 20% of elderly patients ≥ 70 years age was able to tolerate sorafenib at a mean dose intensity > 75% compared to 37% of younger patients (*P* < .001). Patients with a performance status of ECOG 0‐1 were more likely to tolerate sorafenib at a dose intensity > 75% compared with patients ECOG 2‐4 (32% vs 27%, respectively, *P* = .015).

**TABLE 2:** Characteristics of patients comparing dose intensities

Patient Characteristic	<50% n (%) N = 304	50%‐75% n (%) N = 163	>75% n (%) N = 214	*P*
Age, years	65	66	61	<.001
Mean	255 (84%)	34 (21%)	169 (79%)	
≥70	49 (16%)	129 (79%)	45(21%)	
Gender				.718
Male	240 (79%)	133 (82%)	174 (81%)	
Female	64 (11%)	30 (18%)	40 (19%)	
Ethnicity				.185
East‐Asian	123 (40%)	56 (34%)	71 (33%)	
Other	181 (53%)	107 (66%)	143 (67%)	
Liver disease etiology				.189
None	59 (19%)	24 (15%)	34 (16%)	
Hepatitis B	103 (34%)	50 (31%)	69 (32%)	
Hepatitis C	93 (31%)	46 (28%)	56 (26%)	
Alcohol	27 (9%)	29 (18%)	36 (17%)	
NASH	11 (4%)	7 (4%)	14 (6%)	
Other	11 (4%)	7 (4%)	5 (2%)	
Any prior treatment	88 (29%)	47 (29%)	76 (35%)	.224
Liver Resection	76 (25%)	37 (23%)	50 (23%)	.833
Radiofrequency Ablation	78 (26%)	33 (20%)	37 (17%)	.066
Alcohol Injection	12 (4%)	5 (3%)	7 (3%)	.861
Bland Embolization	11 (4%)	4 (2%)	6 (3%)	.755
TACE	115 (38%)	57 (35%)	55 (26%)	.14
TARE	6 (2%)	6 (4%)	10 (5%)	.216
Baseline AFP level				.647
>400	135 (44%)	64 (39%)	89 (42%)	
≤400	150 (49%)	85 (52%)	111 (52%)	
Unknown	19 (6%)	14 (9%)	14 (6%)	
Baseline ECOG performance status				.015
0‐1	257 (84%)	147 (90%)	191 (89%)	
2	40 (13%)	16 (10%)	18 (8%)	
3‐4	4 (1%)	0	4 (2%)	
Baseline liver function (Child‐Pugh)				.648
A	254 (83%)	143 (88%)	188 (88%)	
B	47 (15%)	20 (12%)	25 (12%)	
C	1 (<1%)	0	0	
Baseline metastatic disease				.207
Yes	138 (45%)	71 (43%)	111 (52%)	

Abbreviations: AFP, alpha fetoprotein; ECOG, Eastern Cooperative Oncology Group; NASH, non‐alcoholic steatohepatitis; TACE, trans‐arterial chemoembolization; TARE, trans‐arterial radioembolization.

**FIGURE 2:**
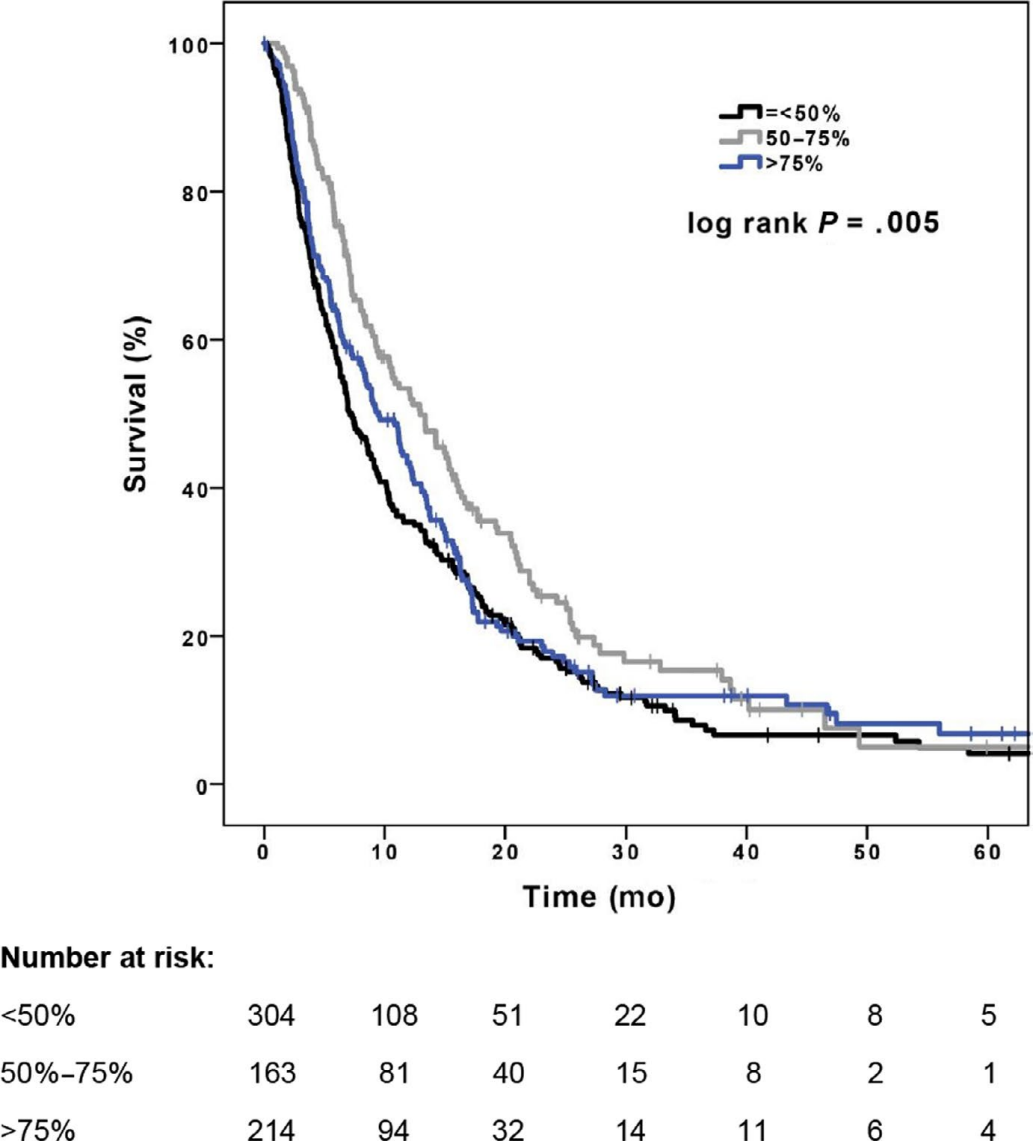
Kaplan‐Meier survival curves for different dose intensities

In the mean dose intensity comparison, the median survival for a mean dose intensity > 75% was 9.5 months (95% CI, 7.5‐11.5 months), 12.9 months for 50%‐75% (95% CI, 9.7‐16.2 months), and 7.1 months for a dose intensity of < 50% (95% CI, 5.9‐8.3 months) (*P* = .005) (Figure 2).

### 3.4 Propensity score analysis

#### 3.4.1 Starting dose comparison

Of the total 681 patients, 243 patients who started on full dose sorafenib were matched to 243 patients who started on half‐dose. Baseline covariates were well‐balanced between the two groups(Appendix Table A, Appendix Figure A.1 and A.2). Previous TARE was the only covariate in which the groups differed in the matched sample by greater than 0.1 (Appendix Table A). However, only a small number of patients (n = 17) had previous TARE and this difference is unlikely to have a significant impact. After propensity matching, starting dose did not have a significant association with OS (HR, 0.80; 95% CI, 0.61‐1.06, *P* = .12).

In a second analysis with propensity score weighting method (IPTW), the entire sample of 681 patients was used, with an excellent balance of covariates (Appendix Table B). In this model, the difference in OS between the two starting dose groups was also not statistically significant (HR, 0.89; 95% CI, 0.79‐1.01) (*P* = .06).

#### 3.4.2 Mean dose intensity comparison

In this analysis, all the 681 patients were included. The propensity score was calculated using the weighting method (IPTW) and the same baseline covariates from starting dose comparison were used and they were well‐balanced between the groups(Appendix Table C). Similar to the previous results, there was also no significant association between the different mean dose intensities and OS: dose intensity of <50% vs >75% (HR, 1.12; 95% CI, 0.94‐1.39) (*P* = .19); dose intensity of 50%‐75% vs >75% (HR, 0.89; 95% CI,0.71‐1.12) (*P* = .33).

### 3.5 Duration of treatment and dose modifications

The median duration of treatment was 2.8 months (1.5 to 6.11, IQR) for patients who started sorafenib at a reduced dose and 3.2 months (1.7 to 7.2, IQR) for the ones who started at full dose (*P* < .001).

Of the 289 patients who started sorafenib at full dose, about 149 (52%) required a dose reduction. Of the patients who started sorafenib on a reduced dose, around 117 (30%) had their dose increased and 76 (19%) had a further dose reduction during treatment. The median duration of sorafenib treatment was 4.5 months (2.6 to 8.8, IQR) for patients who had a dose increase,4.7 months (2.5 to 6.4, IQR) for patients who had a dose decrease and 2 months (0.9 to 3.7, IQR) for the group who did not have any dosage modification (*P* < .001).

In exploratory analysis, the median OS for patients with sorafenib dose increase, decrease and no change were 12.3, 13.3, and 6.3 months respectively (*P* < .001).

### 3.6 Multivariable analysis

**FIGURE 3:**
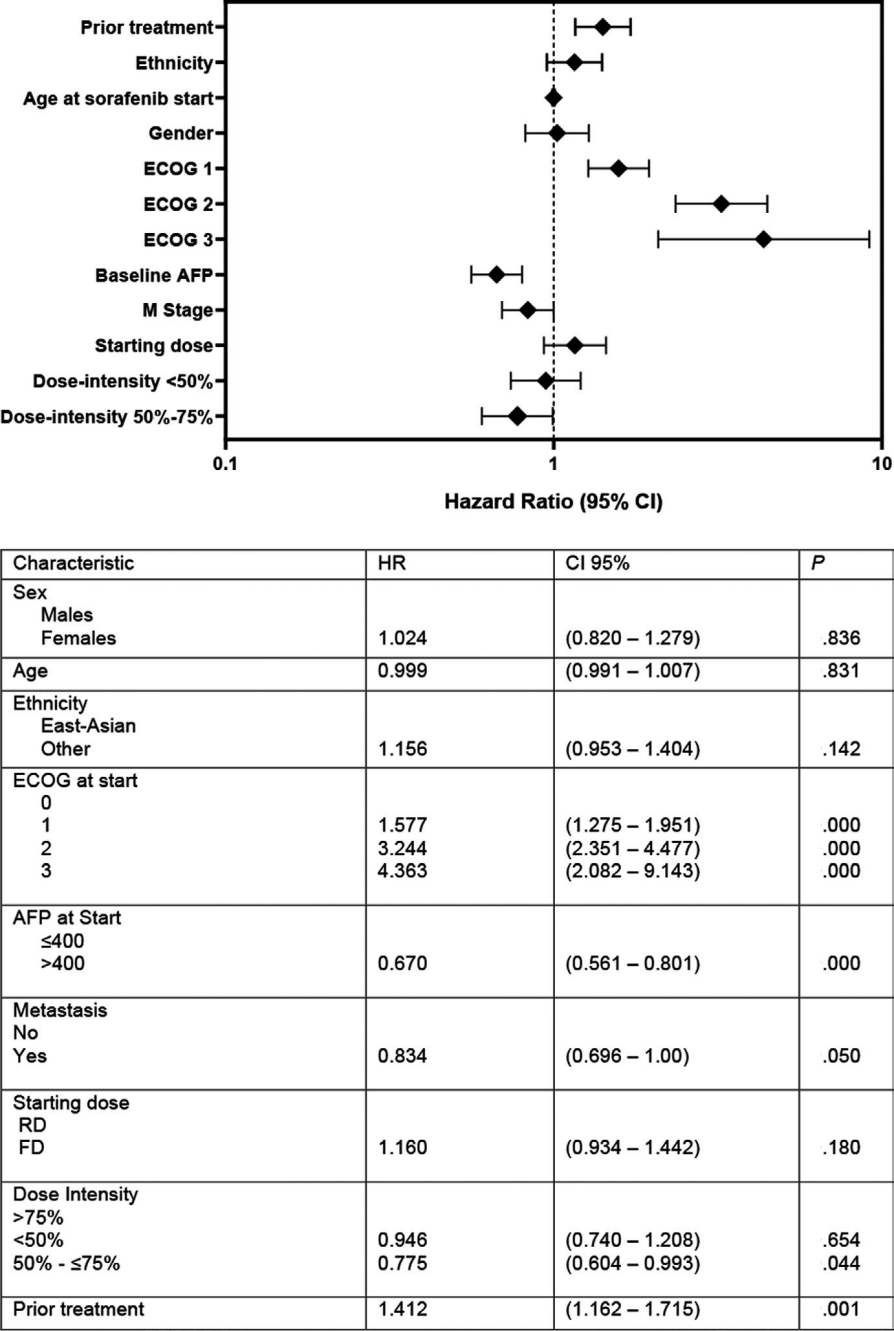
Multivariate analysis of overall survival

In multivariate models that adjusted for other factors including demographic, stage, performance status, AFP and prior treatment, starting dose was not a predictor of survival (HR 1.16, 95% CI 0.93‐1.44, *P* = .18) (Figure 3). Dose intensity of <50% vs >75% was also not considered a predictor of survival (HR 0.95, 95% CI 0.74‐1.21, *P* = .65). Patients who received sorafenib at a dose intensity of 50%‐75% appeared to have better survival outcomes compared to those treated with a dose intensity of >75% (HR 0.77; 95% CI 0.60‐0.99, *P* = .04) (Figure 3).

Two additional multivariable analyses were also performed to evaluate the presence of multicollinearity of starting dose and dose intensity, but all the results were quite similar. In the first model, dose intensity was removed and, in this analysis, survival was not affected by starting dose (HR 1.17, 95% CI 0.97‐1.39, *P* = .10). In the second model with starting dose excluded, dose intensity also did not predict survival (<50% vs >75% [HR 1.03; 95% CI 0.84‐1.27, *P* = .75], 50%‐75% vs >75% [HR 0.79; 95% CI 0.62‐1.01, *P* = .07]).

### 3.7 Adverse events and discontinuation of sorafenib

The majority of patients (86%) experienced at least one adverse event during treatment. The most common adverse events were fatigue (64%), diarrhea (39%), anorexia (36%) and rash (29%) and hand‐foot syndrome (29%). Hypertension occurred in 14% patients (Appendix Table D). The most common reason for discontinuation of treatment was disease progression (66%). About 9% of patients stopped sorafenib based on their own preference and only 19% of patients stopped sorafenib due to toxicities.

The rates of adverse events were quite similar between patients who started sorafenib at a lower dose and those who started with the standard dose (Appendix Table C), with the exception of hand‐foot syndrome (24% vs 35%, respectively, *P* = .002) and mucositis (8% vs 14%, respectively, *P* = .008).

In the mean dose‐intensity comparison, adverse event rates seemed to occur most frequently in the patients who received sorafenib at a dose‐intensity of 50%‐75% (*P* < .001).The most common adverse events were fatigue (74%), rash (37%), hand‐foot syndrome (47%), diarrhea(47%) and anorexia (39%) (Appendix Table C).

There was no relationship between starting sorafenib at full dose vs reduced dose and stopping treatment due to toxicities (*P* = .48) (Table 3). Moreover, there were no significant difference between the different dose intensities and discontinuation of sorafenib (*P* = .25) (Table 3).

**TABLE 3:** Reasons for discontinuation of sorafenib

(A) Starting dose			
	Dose reduced n (%) N = 392	Full dose n (%) N = 289	*P*
Any adverse event	75(19%)	54 (19%)	.481
Patient preference	41 (10%)	22 (7%)	
Disease progression	255 (65%)	192 (66%)	

(B) Dose‐intensity				
	< 50% n (%) N = 304	50%‐75% n (%) N = 163	>75% n (%) N = 214	*P*
Any adverse event	64 (21%)	31 (19%)	34 (16%)	.246
Patient preference	32(10%)	12(7%)	19 (9%)	
Disease progression	182 (60%)	114 (70%)	151 (70%)	

## 4 DISCUSSION

This large multicenter study of HCC patients has confirmed that starting sorafenib at a lower dose compared to the full dose does not affect OS. While patients receiving sorafenib at a dose intensity of 50%‐75% appeared to have superior survival in the univariate analysis, the multivariate and propensity score analysis showed that dose intensity does not affect survival. In addition, there was no relationship between sorafenib starting dose or dose intensity and discontinuation of sorafenib due to adverse events.

The results of the present study are consistent with the findings of other studies examining starting dose of sorafenib and its effect on survival in other populations of HCC patients. Two smaller Japanese studies found comparable PFS and OS in HCC patients started on full or half‐dose sorafenib.^11^, ^12^ The American study of almost exclusively male patients treated at Veterans Affairs hospitals also showed no difference in OS between starting sorafenib at full dose compared to a reduced dose.^13^

As with the American study, we were also able to confirm that there is no difference in rates of sorafenib discontinuation due to adverse events by starting at full dose vs reduced dose. Also, rates of sorafenib discontinuation due to patient preference were not affected by starting dose. These findings are important since the main reason to start sorafenib treatment at a lower dose is to minimize the occurrence and/or severity of adverse events. A single high‐grade adverse event could lead to a treating physician or patient stopping the sorafenib treatment. Multiple lower grade adverse events may also lead to a patient requesting to stop treatment. The results of this study indicate that starting sorafenib at a full or reduced dose does not affect discontinuation rates due to poor tolerability and therefore either starting strategy is reasonable.

Patients started on full dose sorafenib may quickly require significant dose reductions due to adverse events while patients started on a reduced dose of sorafenib may have their dose rapidly escalated in the absence of toxicities. In our data about 30% of the patients who started a reduced dose of sorafenib had their dose escalated during treatment and almost half of the patients who started at full dose required a dose reduction. This is in agreement to what was seen at the SHARP and Asia‐Pacific trials where they reported a dose reduction of sorafenib of about 26% and 30% respectively.^4^, ^5^ Due to these possibilities, starting dose of sorafenib may not be the best parameter to evaluate the correlation between treatment dosage and discontinuation rates/toxicity or mortality. Dose intensity over the full course of sorafenib treatment was hypothesized to have a greater effect on survival than starting dose. In the SOFIA study, patients who received a sorafenib at half‐dose or more for 70% of their treatment had a better OS (21.6 months) compared to the remaining patients who maintained full dose or half‐dose for less than 70% of treatment period (9.6 months) (*P* = .0006).^16^ In their multivariate analysis full dose sorafenib was demonstrated as an independent predictor of mortality.^16^ It is difficult to directly compare these results to our study since the dose intensity categories are different. However, we did find a trend toward increased mortality in patients who were on sorafenib at a mean dose intensity of less than 50%. This is expected since this group had the lowest baseline performance status compared to the other two groups and ultimately they may have been treated with subtherapeutic doses of sorafenib. There was also a trend of patients on sorafenib at a mean dose intensity of 50%‐75% appearing to have better survival compared with patients who had sorafenib at mean dose intensity of > 75% in the univariate analysis. This was despite the 50%‐75% mean dose intensity group experiencing more adverse events than the other groups. More adverse events on sorafenib may actually predict for better outcomes since an Italian study suggested that the occurrence of some angiogenic side effects such as rash, diarrhea and hypertension may predict for better sorafenib effectiveness.^17^ Another possible explanation is that patients in the 50%‐75% group were more likely to receive dose reductions due to drug‐related toxicities thus allowing them to stay on sorafenib for a longer period of time resulting in better survival. While many of the patients who received a mean dose intensity of >75% may not have experienced significant adverse events, some may have refused a dose reduction despite toxicities, stopped treatment sooner and had worse survival as a result.

We found that dose modification of sorafenib, either increasing or reducing the dose to optimal levels, appears to result in a longer duration of treatment and better OS compared to the patients who did not have any dose adjustments. For patients who started sorafenib at a reduced dose the subsequent dose increases were likely due to excellent tolerance and the dose escalation may have resulted in boosting sorafenib to a better therapeutic level. As mentioned above dose reductions may allow patients to have a prolonged exposure to sorafenib which can then result in improved survival. Conversely, patients without dose reductions could have either discontinued sorafenib earlier due to more toxicity or did not have any toxicity due to subtherapeutic drug exposure. Others may have had rapidly declining performance status or liver function and rather than dose reductions instead had sorafenib discontinued.

Patients who started at full dose sorafenib stayed on treatment for a slightly longer time than the ones who started at a reduced dose. There is likely selection bias in that patients who started at a reduced dose were probably less well than those started on full dose. Considering the results of the SOFIA study and this present study it may be reasonable to aim for patients to be treated with an average dose of sorafenib that is equal to or greater than the half‐dose, but not necessarily pushing for the highest dose intensity, especially in elderly or more unwell patients.

While sorafenib was shown to improve overall survival compared to placebo in an unselected population of patients with advanced HCC it is associated with a low response rate and a number of potential toxicities.^4^, ^5^ At this time there are no predictive biomarkers to indicate which patients will benefit from sorafenib. Several studies have attempted to do this by examining baseline characteristics of patients or plasma biomarkers, but most have failed to predict response to sorafenib.^18^, ^19^, ^20^, ^21^, ^22^, ^23^ However, one study was able to show that chronic treatment with metformin was associated with a poorer prognosis for patients who were treated with sorafenib.^24^ Additionally, as previously mentioned, an Italian group created a scoring system that suggested the occurrence of some angiogenic side effects such rash, diarrhea and hypertension may predict better response rates to sorafenib.^17^ There is still a need for prospective studies to validate these results and other studies to better understand HCC biology and response to therapy.

As with many observational studies, this study has some limitations compared to clinical trials, but is likely a better reflection of real‐world clinical practice. With the retrospective collection of data, selection bias was possible despite propensity score matching. Additionally, not all data, especially toxicities, were well‐documented in the patient charts. Also, dose reduction and discontinuation of treatment were at the discretion of the attending physician which could have led to some patients being treated more aggressively, while others treated more conservatively. We should also mention that a clear trend toward improved survival can be seen with the patients treated with sorafenib at a dose intensity of 50%‐75% (Figure 2) and perhaps the lack of statistically significant survival benefit may be a function of the sample size. Lastly, in this study quality of life was not directly evaluated since data were only available regarding toxicities. Quality of life is an important endpoint that should be examined in future prospective studies of tyrosine kinase inhibitors in HCC treatment since the goal of optimal dosing is to improve quality of life while maintaining the survival benefit.

This study has several strengths. Firstly, we collected data from a large number of cancer centers in Canada which gave us a large and representative sample of HCC patients compared to other studies.^11^, ^12^, ^16^ Secondly, as a result of the diversity of the Canadian population, our sample of HCC patients was more heterogenous than prior studies, including 20% females and 37% East Asian patients. Thirdly, these HCC patients were not selected for sorafenib treatment based on strict trial inclusion criteria and, therefore can be considered a better reflection of treating real‐world patients in clinical practice and its complexity.

## 5 FUTURE DIRECTIONS

While sorafenib may be replaced by other first‐line treatments for HCC in the near future, many other newer tyrosine kinase inhibitors are likely to remain in use for the management of HCC. The mechanisms of action and toxicities of lenvatinib, regorafenib, and cabozantinib are similar to sorafenib. It is likely that starting dose and dose intensity will affect survival of HCC patients receiving these newer tyrosine kinase inhibitors in the same way. However, this will need to be confirmed in the future once more patients have been treated with these drugs and their real‐world outcomes can be examined.

In conclusion, this study suggests that the starting dose and dose‐intensity of sorafenib during treatment may not affect survival in patients with HCC. The rate of discontinuation of therapy due to adverse events or patient preference also did not seem to be affected by these dosing characteristics of sorafenib.

## CONFLICT OF INTEREST

Vincent C Tam ‐ Honoraria/Advisory Boards: BMS, Celgene, Eisai, Ipsen; Research Support (To Institution): Bayer, Eisai. Jennifer J Knox ‐ advisory role: Merck, BMS, Pfizer. Grant and research support: Merck, Pfizer, Astra Zeneca, Ipsen. Winson Y. Cheung‐ Received honoraria and research funding from Roche. Richard Lee‐Ying ‐ Advisory board for Bayer, Eisai, Roche, Janssen, Merck, Celgene, Sanofi Speaker for Janssen Research funding from Sanofi. Hao‐Wen Sim ‐ Research funding (To Institution): AbbVie, Bristol‐Myers Squibb. The other authors have no conflict of interest.

## AUTHOR CONTRIBUTIONS

**Conception and design:** Vincent C. Tam. **Collection and assembly of data:** Mohammed A. Alghamdi,Richard Lee‐Ying, Hao‐Wen Sim, Haider Samwi, Jennifer J. Knox, Kelvin K. Chan, Yoo‐Joung Ko, Mina Swiha, Eugene Batuyong, Adriana Romagnino, Winson Y. Cheung. **Data analysis and interpretation:** Mohammed A. Alghamdi, Carla P. Amaro, Richard Lee‐Ying,Kelvin K. Chan, Vincent C. Tam. **Manuscript writing:** Mohammed A. Alghamdi, Carla P. Amaro, Richard Lee‐Ying, Vincent C. Tam. **Final approval of manuscript**: All authors. **Accountable for all aspects of the work**: All authors.

## Supporting information

Supplementary Material

## Data Availability

The data that support the findings of this study are available on request from the corresponding author. The data are not publicly available due to privacy or ethical restrictions.
