# Supplementary material for: Effect of sorafenib starting dose and dose intensity on survival in patients with hepatocellular carcinoma: Results from a Canadian Multicenter Database
Source: Cancer Med. 2020 Jun 11;9(14):4918–28. doi: 10.1002/cam4.3228 (PMC7367626; doi:10.1002/cam4.3228)
Supplement: Supplementary file 1 — Supplementary Material [file CAM4-9-4918-s001.pdf]

Appendix Table A: Standardized differences of baseline covariates in original and matched sample

	Original sample (n=681)			Matched sample (n=486)		
**Variable**	**Reduced dose N=392**	**Full dose N=289**	**Std diff**	**Reduced dose N=243**	**Full dose N=243**	**Std diff**
**Age**	65.5±12.1 66 (58, 74)	62.1±11.1 63 (57, 70)	0.295	62.7±11.8 63 (56, 71)	62.9±10.9 64 (58, 71)	0.016
**Female**	87 (22.2)	47 (16.3)	0.151	43 (17.7)	44 (18.1%)	0.011
**Ethnicity (Other vs rest*)**	234 (59.7)	197 (68.2)	0.177	159 (65.4)	159 (65.4)	0.000
**Hepatitis B**	136 (34.7)	93 (32.2)	0.053	81 (33.3)	78 (32.1)	0.026
**Hepatitis C**	124 (31.6)	85 (29.4)	0.048	76 (31.3)	73 (30.0)	0.027
**Alcohol related**	84 (21.4)	75 (26.0)	0.107	62 (25.5)	61 (25.1)	0.009
**None Etiology**	65 (16.6)	47 (16.3)	0.009	39 (16.1)	42 (17.3)	0.033
**Liver cirrhosis**	275 (70.2)	205 (70.9)	0.017	170 (70.0)	171 (70.4)	0.009
**ECOG PS (0/1 vs rest*)**	344 (78.8)	251 (86.9)	0.027	215 (88.5)	207 (85.2)	0.097
**Child Pugh (A vs rest*)**	330 (84.2)	255 (88.2)	0.118	215 (88.5)	210 (86.4)	0.062
**PVT (yes vs rest*)**	159 (40.6)	113 (39.1)	0.030	101 (41.6)	94 (38.7)	0.059
**T stage (1/2 vs rest*)**	139 (35.5)	86 (29.8)	0.122	74 (30.5)	77 (31.7)	0.027
**N stage (N0 vs rest*)**	263 (67.1)	186 (64.4)	0.058	159 (65.4)	155 (63.8)	0.034
**M stage (M0 vs rest*)**	200 (51.0)	152 (52.6)	0.032	129 (53.1)	124 (51.0)	0.041
**Tumor extension (>50% vs rest*)**	113 (28.8)	93 (32.2)	0.073	81 (33.3)	77 (31.7)	0.035
**Liver resection**	89 (22.7)	74 (25.6)	0.068	63 (25.9)	54 (22.2)	0.087
**Tumor ablation**	98 (25.0)	50 (17.3)	0.189	47 (19.3)	43 (17.7)	0.042
**TACE**	156 (39.8)	71 (24.6)	0.330	66 (27.2)	68 (28.0)	0.018
**TARE**	8 (2.0)	14 (4.8)	0.154	6 (2.5)	11 (4.5)	0.112
**SBRT**	31 (7.9)	6 (2.1)	0.270	6 (2.5)	6 (2.5)	0.000
**Alcohol Injection**	15 (3.8)	9 (3.1)	0.039	7 (2.9)	7 (2.9)	0.000
**Liver transplant**	21 (5.4)	12 (4.2)	0.057	11 (4.5)	10 (4.1)	0.020
Abbreviations: ECOG PS, Eastern Cooperative Oncology Group performance status; PVT, portal vein thrombosis; SBRT, stereotactic body radiation; Std diff, Standardized difference; TACE, trans-arterial chemoembolization; TARE, trans-arterial radioembolization;						
*Unknown were included under the rest group.						

Appendix Figure A.1: Distribution of PS in the original sample before matching

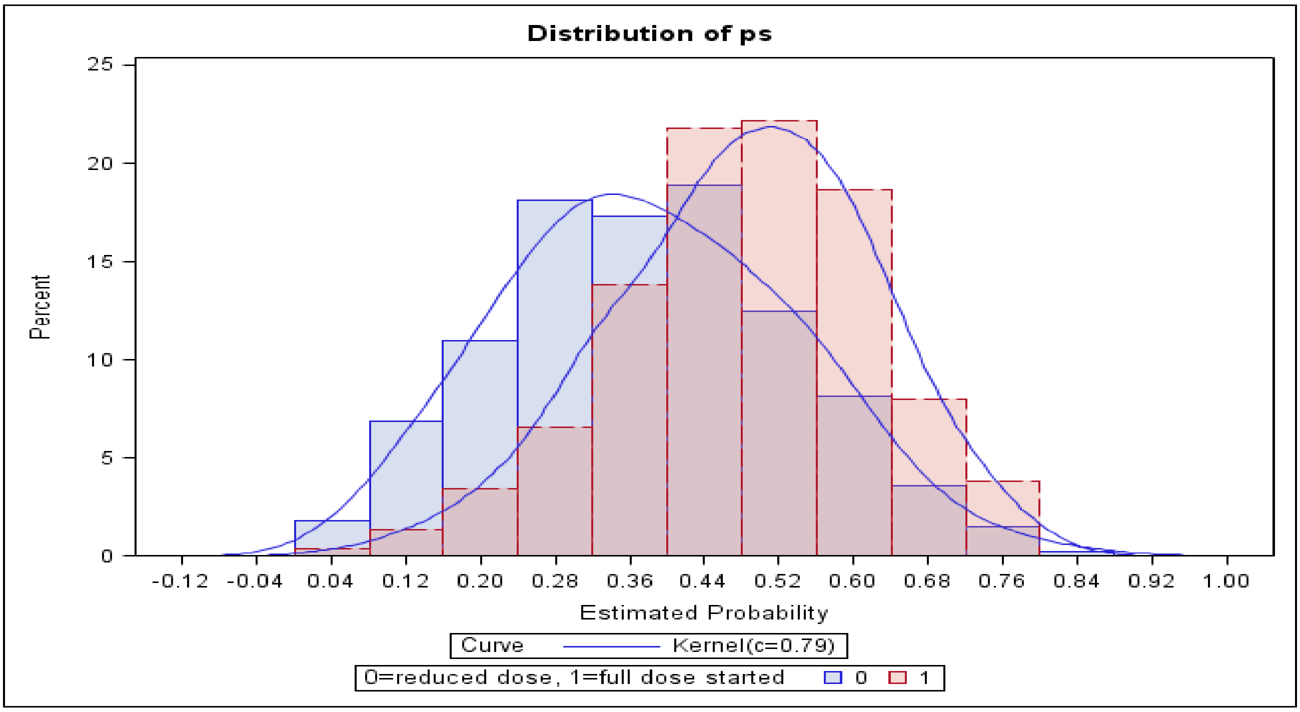

Appendix Figure A.2: Distribution of PS in the matched sample

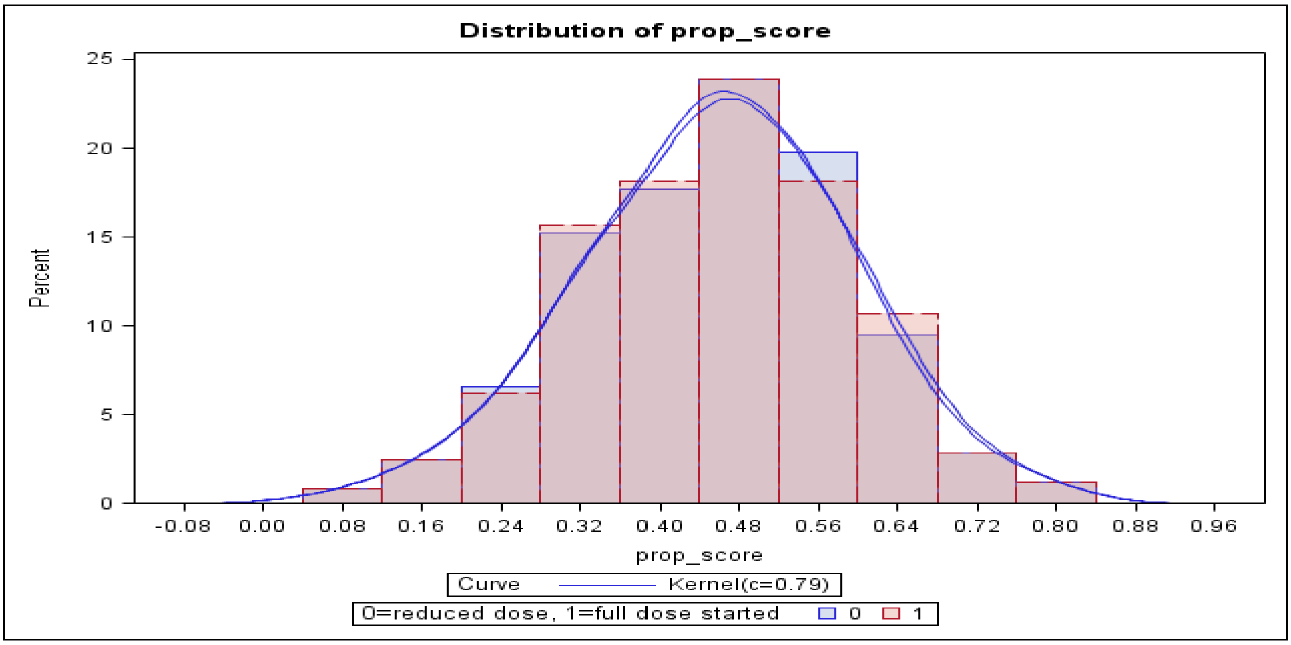

Appendix Table B: Standardized differences in the weighted IPTW sample

Variable	Std diff
**Age**	0.0174
**Female**	0.0146
**Ethnicity**	0.0116
**Hepatitis B**	0.0075
**Hepatitis C**	0.0284
**Alcohol related**	0.0056
**None Etiology**	0.0058
**Liver cirrhosis**	0.0018
**ECOG 0/1**	0.0003
**Child Pugh A**	0.0314
**PVT (yes)**	0.0295
**T stage (1/2)**	0.0122
**N stage (N0)**	0.0122
**M stage (M0)**	0.0162
**Tumor extension (>50%)**	0.0162
**Liver resection**	0.0150
**Tumor ablation**	0.0327
**TACE**	0.0151
**TARE**	0.0067
**SBRT**	0.0454
**Alcohol Injection**	0.0182
**Liver transplant**	0.0210

Appendix Table C: Comparison between unadjusted and adjusted weighted IPTW sample

	<50% (n=304)	50-75% (n=163)	>75% (n=214)	Chisq p	IPTW Weighted <50%	IPTW Weighted 50-75%	IPTW Weighted >75%	IPTW weighted chisq p
**Age (median)**	65	66	62	<.001	64	64	64	0.97
**female**	64 (21.1)	30 (18.4)	40 (18.7)	0.72	20.4	20.2	21.7	0.91
**Ethnicity (other vs rest)**	181 (59.5)	107 (65.6)	143 (66.8)	0.18	64.1	61.7	63.8	0.86
**Hepatitis B**	106 (34.9)	53 (32.5)	70 (32.7)	0.83	33.0	34.1	34.2	0.95
**Hepatitis C**	99 (32.6)	50 (30.7)	60 (28.0)	0.55	31.4	30.1	31.3	0.96
**Alcohol related**	55 (18.1)	48 (29.5)	56 (26.2)	0.01	23.2	23.5	23.6	0.99
**None Etiology**	56 (18.4)	23 (14.1)	33 (15.4)	0.43	16.8	15.8	15.2	0.88
**Liver cirrhosis**	203 (66.8)	128 (78.5)	149 (69.6)	0.03	69.9	71.5	70.0	0.92
**ECGO PS (0/1 vs rest)**	257 (84.4)	147 (90.2)	191 (89.3)	0.13	87.3	87.7	87.3	0.99
**Child Pugh (A vs rest)**	254 (83.6)	143 (87.7)	188 (87.9)	0.29	85.6	86.1	86.0	0.98
**PVT (yes vs rest)**	118 (38.8)	59 (36.2)	95 (44.4)	0.24	39.5	38.4	41.2	0.85
**Tstage (1/2 vs rest)**	108 (35.5)	53 (32.5)	64 (29.9)	0.40	32.8	35.6	31.5	0.70
**Nstage (N0 vs rest)**	207 (68.1)	109 (66.9)	133 (62.2)	0.36	67.4	64.5	66.8	0.81
**Mstage (M0 vs rest)**	159 (52.3)	92 (56.4)	101 (47.2)	0.20	50.9	50.1	50.1	0.98
**Tumor extension (>50% vs rest)**	97 (31.9)	45 (27.6)	64 (29.9)	0.62	30.3	28.5	32.6	0.68
**Liver resection**	76 (25.0)	37 (22.7)	50 (23.4)	0.83	24.1	24.9	23.8	0.96
**Tumor ablation**	78 (25.7)	33 (20.3)	37 (17.3)	0.07	21.3	22.2	21.6	0.97
**TACE**	115 (37.8)	57 (35.0)	55 (25.7)	0.014	33.2	34.0	32.8	0.97
**TARE**	6 (2.0)	6 (3.7)	10 (4.7)	0.22	3.7	3.5	3.5	0.99

**SBRT**	21 (6.9)	9 (5.5)	7 (3.3)	0.20	5.7	5.3	6.5	0.88
**Alcohol Injection**	12 (4.0)	5 (3.1)	7 (3.3)	0.86	3.1	3.0	2.9	0.99
**Liver transplant**	1. (4.3)	10 (6.1)	10 (4.7)	0.67	4.4	5.0	4.6	0.96
Abbreviations: ECOG PS, Eastern Cooperative Oncology Group performance status; PVT, portal vein thrombosis; SBRT, stereotactic body radiation; Std diff, Standardized difference; TACE, trans-arterial chemoembolization; TARE, trans-arterial radioembolization;								

Appendix Table D: Observed adverse events

(A) Starting dose

Adverse Event	Dose reduced n (%) N=392	Full dose n (%) N=289	P
**Any Toxicity**	329 (84%)	257 (89%)	0.063
**Hypertension**	55 (14%)	40 (14%)	0.944
**Fatigue**	242 (62%)	191 (66%)	0.243
**Rash**	106 (27%)	91 (31%)	0.206
**Hand-foot syndrome**	95 (24%)	102 (35%)	0.002
**Headache**	11 (3%)	7 (2%)	0.758
**Diarrhea**	150 (38%)	116 (40%)	0.620
**Mucositis**	31 (8%)	41 (14%)	0.008
**Alopecia**	29 (7%)	27 (9%)	0.361
**Weight loss**	97 (25%)	84 (29%)	0.207
**Anorexia**	144 (37%)	99 (34%)	0.505

(B) Dose-intensity

Adverse Event	< 50% n (%) N=304	50-75% n (%) N=163	>75 % n (%) N= 214	*P*
**Any Toxicity**	242 (80%)	159 (97%)	185 (86%)	<0.001
**Hypertension**	38 (12%)	28 (17%)	29 (13%)	0.372
**Fatigue**	177 (58%)	120 (74%)	136 (63%)	0.004
**Rash**	82 (27%)	60 (37%)	55 (26%)	0.037
**Hand-foot syndrome**	67 (22%)	77 (47%)	53 (25%)	<0.001
**Headache**	8 (3%)	8 (5%)	2 (1%)	0.059
**Diarrhea**	101(33%)	77 (47%)	88 (41%)	0.009
**Mucositis**	27 (9%)	26 (16%)	19 (9%)	0.038
**Alopecia**	11 (4%)	24 (15%)	21 (10%)	<0.001
**Weight loss**	65 (21%)	58 (36%)	58 (27%)	0.004
**Anorexia**	112 (37%)	63 (39%)	68 (32%)	0.328
